# Supplementary material for: Sulfur-mediated chalcogen versus hydrogen bonds in proteins: a see-saw effect in the conformational space
Source: QRB Discov. 2023 Apr 27;4:e5. doi: 10.1017/qrd.2023.3 (PMC10411326; doi:10.1017/qrd.2023.3)
Supplement: Supplementary file 1 [file S2633289223000030sup001.docx]

Supplementary Information

**Sulfur-mediated chalcogen versus hydrogen bonds in proteins:**

**a seesaw effect in the conformational space**

Vishal Annasaheb Adhav^1^, Sanket Satish Shelke^1^, P. Balanarayan^2^,

Kayarat Saikrishnan^1*^

^1^Department of Biology, Indian Institute of Science Education and Research, Pune, 411008, India.

^2^Department of Chemical Sciences, Indian Institute of Science Education and Research, Mohali, 140306, India.

^*^Correspondence and requests for materials should be addressed to K.S. (email: saikrishnan@iiserpune.ac.in)

Figure S1. Definition of *ζ* along with some of the representative examples where *ζ* value was greater than 240° or less than 120°, which indicated that the respective H were pointing away from lone-pair region of S.


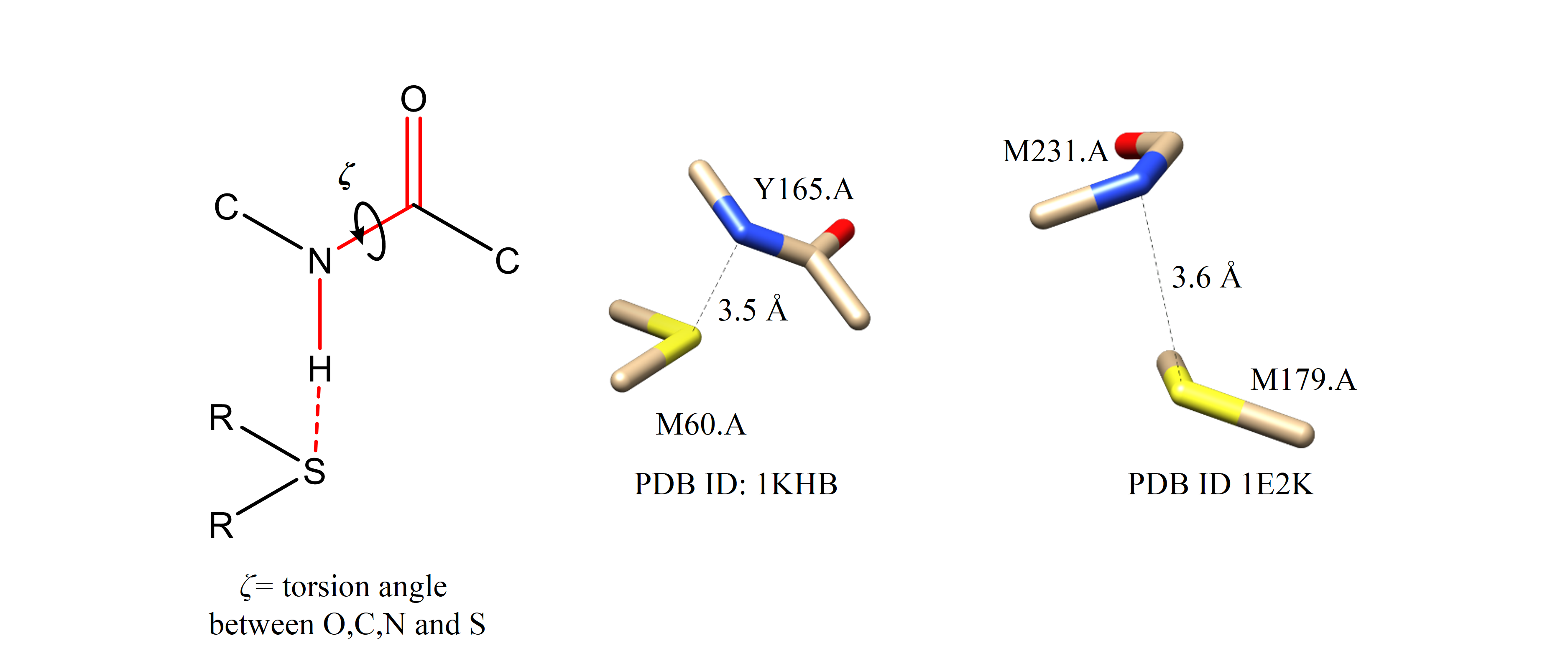


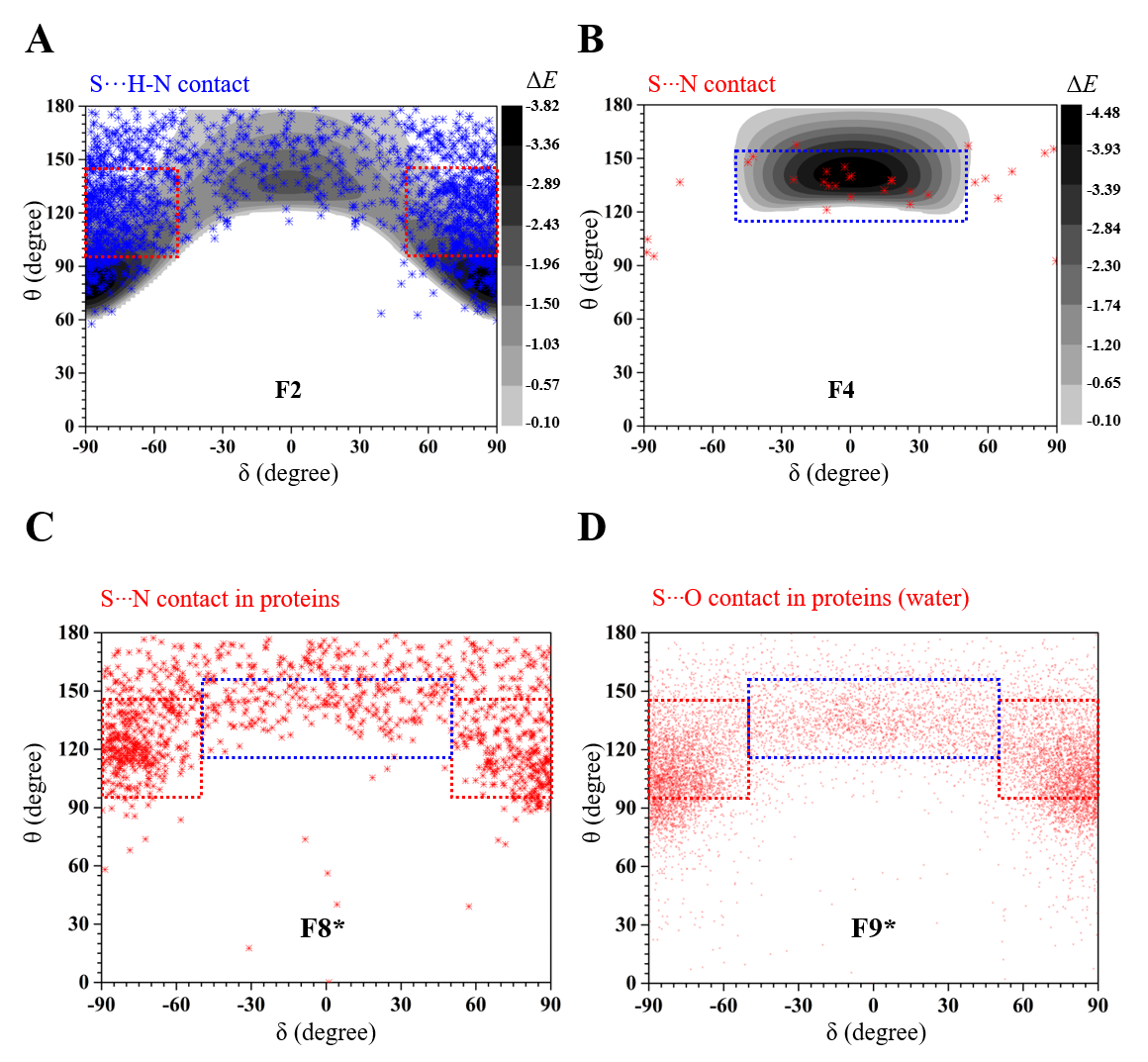
Figure S2. (**A**) Mapping of *θ* and *δ* values of S···H-N contacts in fragment F2 with computationally calculated ΔEs in the background. (**B**) Mapping of *θ* and *δ* values of S···N contacts in fragment F4 with computationally calculated ΔEs in the background. (**C**) S···N and (**D**) S···O contacts formed by methionine and cystine in fragments F8 and F9, respectively. In case of proteins, interacting atoms were separated by at least 6 covalent bonds.

Figure S3. (**A**) Representative examples of H- and Ch-bond in F1-F5 with their CSD ID. In case of fragment F5, *d_S···H_* is marked by an asterisk. Note that H-O/N groups that formed Ch-bond with S in F5 could form H-bond with a neighboring acceptor atom (**B**) Representative example of outliers for S···H-O contacts in F1 (**C**) Representative example of outliers of S···O contacts in F3 from those clustered around at *θ* =75˚ and *δ* =90˚ or -90˚ (refer Figure 1e) and (**d**) Representative examples of outliers of S···O contacts in fragment F3. All the examples are shown with their CSD ID.


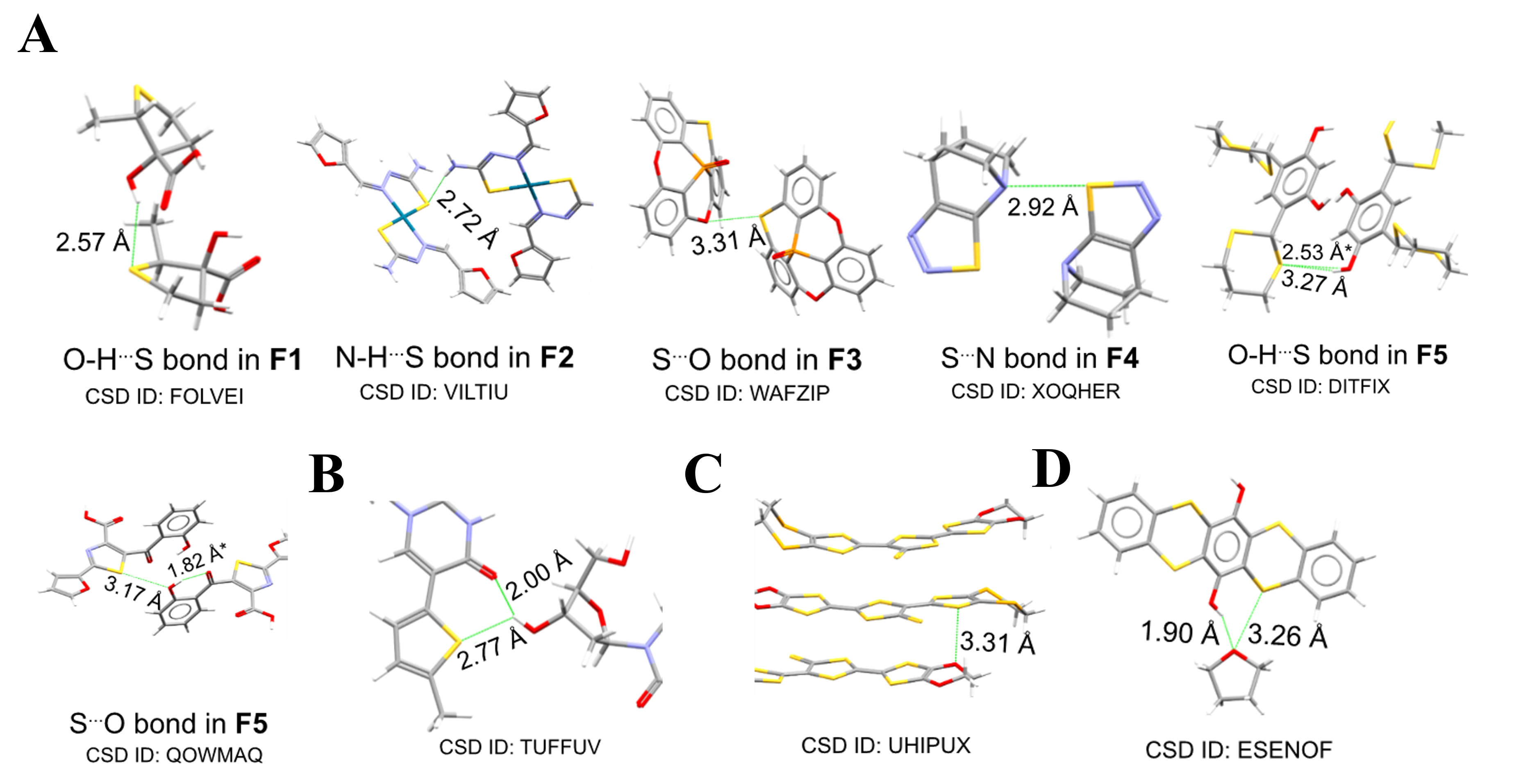


Figure S4. Representative examples of methionine-mediated **(A)** Ch-bond **(B)** H-bond in fragment F7. Representative examples of cystine-mediated **(C)** Ch-bond **(D)** H-bond. Potential coexisting H-bonds are also illustrated. Representative examples of methionine-mediated **(E)** Ch-bond **(F)** H-bond in fragment F8 (**G**) Representative example of cystine-mediated Ch-bond in F8. All structures are shown with their PDB IDs.


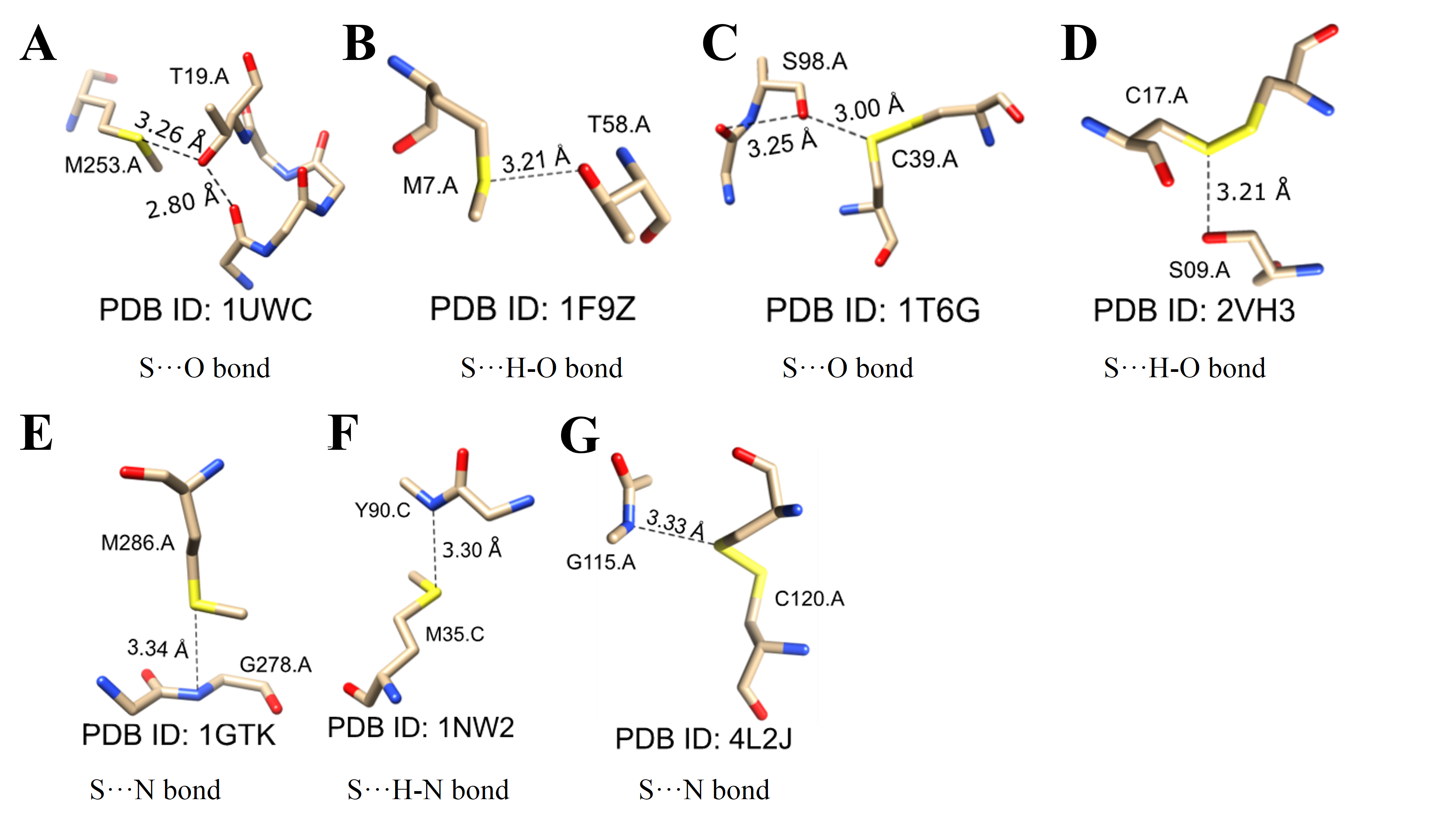


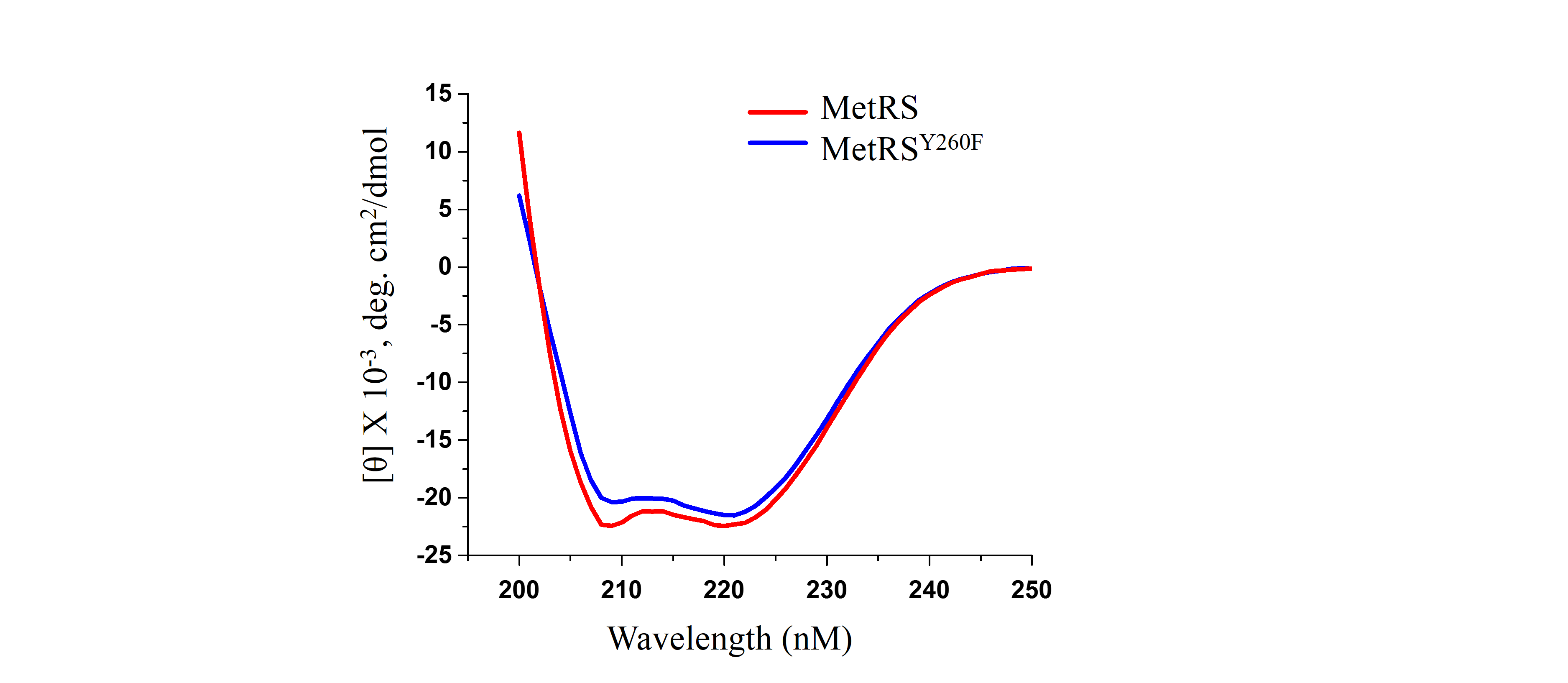


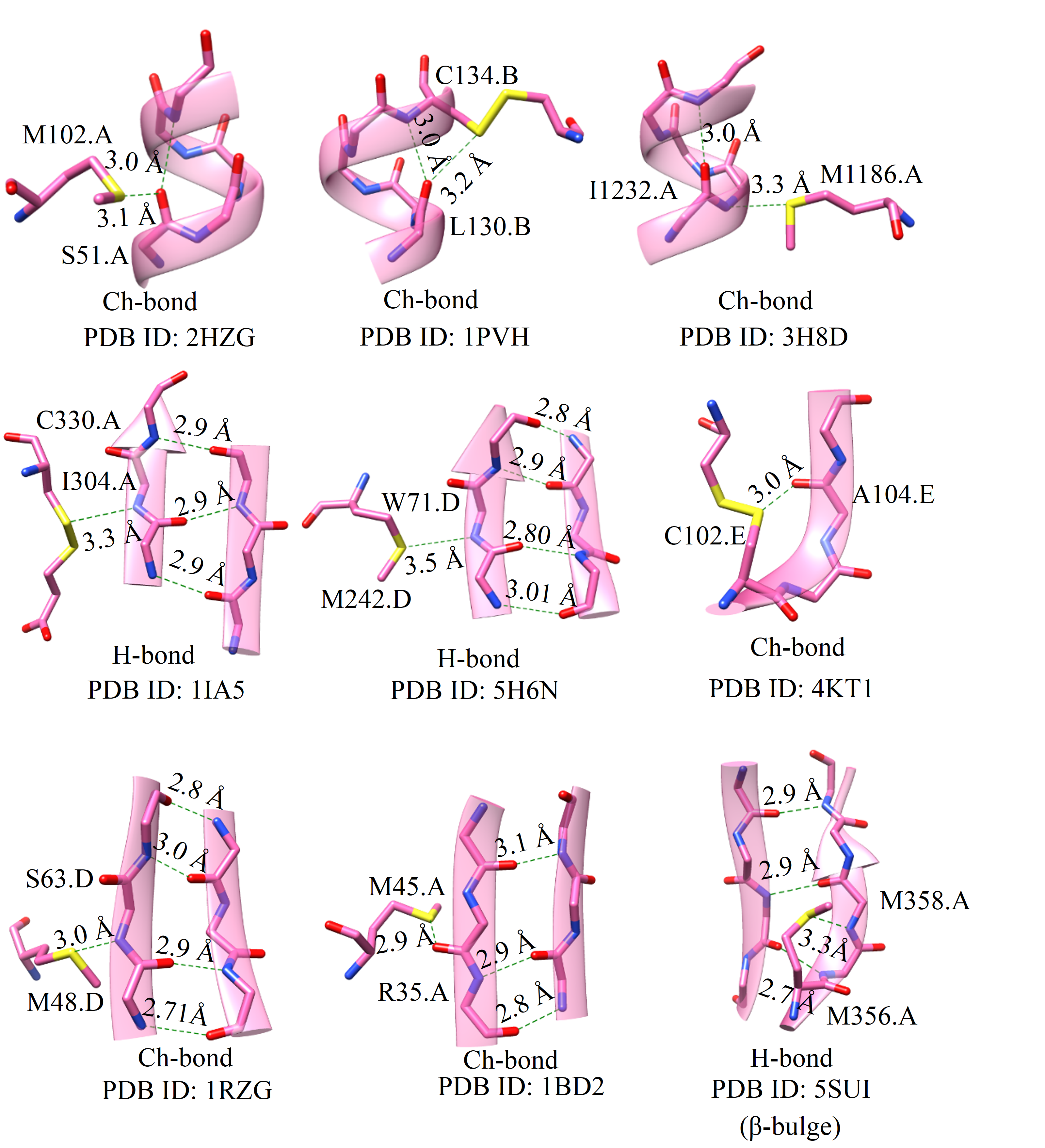


Figure S5. Representative examples of H-bonds and Ch-bonds formed by Met-S^δ^ and Cys-S^γ^ with residues in α-helix and β-sheet. Also, an example of H-bond formed by Met-S^δ^ introducing β-bulge (PDB ID: 5SUI).

Table S1. Classification of the CSD data based on electronic nature of S. S-mediated H-bonds and Ch-bonds were identified using distance (*d*) and angular criteria (*θ* and *δ*) defined in the text.

| Fragment | S···H-O contacts (*N_c_*) | S···H-N contacts (*N_c_*) | S···O contacts (*N_c_*) | S···N contacts (*N_c_*) | Total Fragments  (*N_f_*) |
| --- | --- | --- | --- | --- | --- |
| M-S-M | 49 | 158 | 1 | 0 | 208 |
| M-S-Y | 253 | 507 | 17 | 3 | 780 |
| R-S-R | 48 | 41 | 71 | 12 | 172 |
| E-S-Y | 60 | 56 | 302 | 18 | 436 |
| S (Ar) | 17 | 32 | 318 | 26 | 393 |
| Total | 427 | 794 | 709 | 59 | 1989 |

Table S2. A summary of the number of contacts seen in PDB where *d_S···O_* and *d_S···N_* were below 3.32 Å and 3.35 Å, respectively.

| Interaction | Interacting residues | Number of contacts (*N_c_*) |
| --- | --- | --- |
| S···O contact | S(Ar) and Peptide backbone | 98 |
|  | S(Ar) and Glu/Gln/Asn/Asp | 31 |
|  | S(Ar) and Ser/Thr/Tyr | 46 |
|  | S(Ar) and H_2_O | 147 |
|  | Cystine and Peptide backbone | 2074 |
|  | Cystine and Glu/Gln/Asn/Asp | 245 |
|  | Cystine and Ser/Thr/Tyr | 285 |
|  | Cystine and H_2_O | 980 |
|  | Methionine and Peptide backbone | 2666 |
|  | Methionine and Glu/Gln/Asn/Asp | 727 |
|  | Methionine and Ser/Thr/Tyr | 1320 |
|  | Methionine and H_2_O | 8737 |
|  | Metal-chelated cysteine and Peptide backbone | 69 |
|  | Metal-chelated cysteine and Glu/Gln/Asn/Asp | 148 |
|  | Metal-chelated cysteine and Ser/Thr/Tyr | 369 |
|  | Metal-chelated cysteine and H_2_O | 1552 |
| S···N contact | S(Ar) and Peptide backbone | 13 |
|  | S(Ar) and Arg/His/Lys | 45 |
|  | S(Ar) and Trp/Asn/Gln | 16 |
|  | Cystine and Peptide backbone | 228 |
|  | Cystine and Arg/His/Lys | 212 |
|  | Cystine and Trp/Asn/Gln | 100 |
|  | Methionine and Peptide backbone | 1005 |
|  | Methionine and Arg/His/Lys | 1163 |
|  | Methionine and Trp/Asn/Gln | 719 |
|  | Metal-chelated cysteine and Peptide backbone | 1803 |
|  | Metal-chelated cysteine and Arg/His/Lys | 630 |
|  | Metal-chelated cysteine and Trp/Asn/Gln | 90 |

Table S3. Classification of the PDB data based on electronic nature of S. H-bonds and Ch-bonds were identified using the angular range of *θ* and *δ* defined in the text.

| Fragment | S···H-O contacts (*N_c_*) | S···H-N contacts (*N_c_*) | S···O  contacts (*N_c_*) | S···N  contacts (*N_c_*) | Total Fragments  (*N_f_*) |
| --- | --- | --- | --- | --- | --- |
| M-S-C | 1057 | 1351 | 186 | 98 | 2692 |
| C-S-C | 5354 | 1263 | 4281 | 750 | 11648 |
| C-S-S | 295 | 132 | 2277 | 106 | 2810 |
| S (Ar) | 30 | 41 | 198 | 18 | 287 |
| Total | 6736 | 2787 | 6942 | 972 | 17437 |

Table S4. A summary of the results of the PDB analysis performed to identify H-bond and Ch-bond formed by Cys-S^γ^ or Met-S^δ^ that cap α-helices in proteins.

| Fragment  (residue) | Total Number of α- helix capping contacts (N_T_) ^[a]^ | N-terminal α-helix capping  contacts (N_N_) ^[b]^ | C-terminal α-helix capping  contacts (N_C_) ^[c]^ |
| --- | --- | --- | --- |
| C-S-S  (cystine) | 164 | 42 (25) | 123 (75) |
| C-S-C  (Methionine) | 249 | 138 (37) | 232 (63) |
| C-S-M  (Metal-chelated cysteine) | 1069 | 1068 (100) | 1 (0) |

^[a]^Total number of S···O/ S···H-N contacts found capping α-helices. ^[b]^Total number of S···H-N contacts found capping the N-termini of α-helices. ^[c]^Number of S···O contacts found capping the C-termini of α-helices. The percentage in parenthesis was calculated using [(N_N/C_/ N_T_) x100].

Table S5. A summary of the results of PDB analysis performed to identify H-bond and Ch-bond formed by Cys-S^γ^ or Met-S^δ^ with residues in α-helix (only internal residues) and β-sheets.

| Fragment (residue) | S···H-N interaction | | S···O interaction | | S···N interaction | |
| --- | --- | --- | --- | --- | --- | --- |
|  | α-helix | β- strand | α-helix | β- strand | α-helix | β- strand |
| C-S-S  (Cystine) | 0 | 7 | 297 | 42 | 0 | 3 |
| C-S-C  (Methionine) | 1 | 335 | 156 | 229 | 34 | 34 |
